# Improving the Science of Measles Prevention—Will It Make for a Better Immunization Program?

**DOI:** 10.1371/journal.pmed.1002145

**Published:** 2016-10-11

**Authors:** Julie Garon, Walter Orenstein

**Affiliations:** Division of Infectious Diseases, Emory University School of Medicine, Atlanta, Georgia, United States of America

## Abstract

In a Perspective, Julie Garon and Walter Orenstein discuss Lessler and colleagues' modeling study on measles vaccination and the implications for triggered and routine immunization programs.

In the early 1960s before measles vaccine was introduced, more than 2 million persons died annually from measles [1]. Control efforts reduced that number to an estimated 114,900 deaths in 2014, and many countries have interrupted transmission, achieving elimination of indigenous measles [2]. Yet measles continues to circulate in many countries. A major reason for this is that the measles virus is highly infectious and herd immunity thresholds needed to stop transmission have been estimated to be as high as 94% [3]. A single dose of measles vaccine administered at age 9 mo, the standard age for first vaccination in most developing countries, induces immunity in about 85% of vaccinated children [4]. Over 90% immunity is achieved when the first dose is administered after the first birthday [4]. Because the likelihood of reaching herd immunity thresholds is limited, even with 100% immunization coverage, a second dose was recommended, which seroconverts more than 90% of those who fail to seroconvert after the first dose [5].

When high coverage in routine immunization programs is achieved with two doses, herd immunity thresholds can be reached and transmission stopped. But because routine immunization programs, particularly in developing countries, have not been able to achieve high enough coverage with the recommended two doses of measles-containing vaccines, measles vaccination through mass campaigns, known as supplemental immunization activities (SIAs), have been part of the measles elimination strategy in much of the world. SIAs are periodic mass campaigns in which all children of a defined age range (usually children aged 9 mo to 4 y) are vaccinated at one time regardless of vaccination status. Periodic SIAs are designed to ensure that children without protective immunity are reached by campaigns boosting population immunity. The Global Measles & Rubella Strategic Plan 2012–2020 builds upon successes of previous measles control efforts outlining an elimination strategy focusing on maintaining high levels of population immunity, strengthening surveillance, and responding rapidly to outbreaks [6]. In addition to SIAs occurring at intervals determined by routine coverage levels, reactive response vaccination is recommended for outbreaks in measles mortality reduction areas and in elimination settings [7]. In this issue of *PLOS Medicine*, Justin Lessler and colleagues model the potential impact of triggered campaigns [8], that is, SIAs initiated when a case threshold or percent susceptibility based on serological surveys is reached, instead of fixing an interval for SIAs based on overall routine immunization coverage.

Lessler and colleagues carried out simulations in four settings with different levels of measles incidence [8] and report that SIAs triggered by disease outbreaks could prevent 28,613 cases (95% CI 25,722–31,505) over 15 y in high-incidence settings and 599 cases (95% CI 464–735) in the lowest-incidence setting tested. SIAs triggered by serological surveys, in contrast, could prevent 89,173 cases (95% CI 86,768–91,577) and 744 (95% CI 612–876) cases in the highest- and lowest-incidence settings, respectively, but would be triggered annually in high-incidence settings. The methods used by Lessler and colleagues are well thought through, and the benefits to measles prevention, even when only 20% of the susceptible children are reached with triggered SIAs, well documented. However, outbreak response and emergency SIAs can be disruptive. For example, staff are often diverted from routine activities to implement an SIA. Vaccine supply must be secured, potentially on an urgent basis, requiring financing, shipping, and other logistical preparations. Emergency clinics may need to be established, and special communication efforts must be made to encourage caregivers to bring their children into clinics for vaccination. An economic analysis of the triggered SIA approach would provide a more complete picture and might show that triggered SIAs enhance or impede efforts to improve the overall immunization system, with implications far beyond measles prevention.

While serosurveillance may anticipate potential outbreaks and actions such as triggered campaigns prevent cases of measles, perhaps a greater benefit to the population can be obtained if efforts to control or eliminate measles can be used to strengthen the overall immunization system. This does two things. First, the need for special SIAs may be eventually avoided because the two needed doses of measles vaccine are delivered on a routine basis to enough of the population to reach and surpass herd immunity thresholds. Second, by strengthening routine immunization, not only are measles cases prevented, but potentially many other vaccine preventable diseases are avoided as well.

For example, in the United States, a focus on measles was used to build the overall immunization system. Measles vaccine, introduced in 1963, led to major reductions in incidence of disease. However, a resurgence of measles occurred in 1977, with 57,345 cases reported [9]. The outbreak affected a substantial number of older children and adolescents who had missed vaccination and, due to decreased incidence of cases from effective control interventions, had not been exposed to natural measles virus [9]. The outbreak drove school entry vaccination requirements that, by 1981, had grown to include all 50 states for measles and multiple other vaccines. Another resurgence took place during 1989–1991, when over 53,000 cases, 11,000 hospitalizations, and 123 deaths occurred [9,10]. Although some college students were also affected, the focus of this outbreak was in the unvaccinated preschool population [10].

The US immunization program responded by improving access to all recommended immunizations (including enhanced capability for measuring childhood immunization status), improving management of immunization services and in 1989 implementing a two-dose immunization schedule in response to instances of primary vaccine failure of one dose [9,11]. These measles resurgences served as indicators for shortcomings in the overall immunization program and acted as an impetus for change, leading to measles elimination in the US in the year 2000 [11]. Additionally, the cyclical nature of measles periodically “resets” the social norms for acceptable levels of disease and triggers new and innovative interventions [12]. For example, the 2015 measles outbreak had fewer than 1,000 cases, minor compared to previous resurgences. Yet public outcry about the outbreak gave way to stricter vaccine exemption laws in California, with many other states reconsidering policies [13]. For this reason, measles elimination programs lend themselves to continual strengthening of overall immunization programs, with benefits beyond measles control itself.

The Global Vaccine Action Plan 2011–2020 highlights the need for disease-specific programs such as measles elimination to place efforts into building routine immunization systems [14]. Moving away from independent, “vertical” operation and focusing on integration with national programs speaks to country ownership, shared responsibility, partnership, and sustainability, guiding principles of the plan. Measles programs provide an opportunity to institute a “diagonal” approach [12], in which it is possible to leverage resources available to vertical programs such as measles to deliver other health services and strengthen overall health systems. While implementing additional SIAs based on trigger indicators may provide a scientific advantage, focusing on strengthening overall routine immunization programs will have lasting benefits beyond measles alone.

## Abbreviations

SIA: supplemental immunization activity
